# Investigation of [^11^C]carfentanil for mu opioid receptor quantification in the rat brain

**DOI:** 10.1038/s41598-024-66144-4

**Published:** 2024-07-15

**Authors:** Andrew C. Kelleher, Torben D. Pearson, Joseph Ramsey, Wenjing Zhao, Kelly A. O’Conor, Abolghasem Bakhoda, Tyler Stodden, Min Guo, Seth M. Eisenberg, Sarthak V. Shah, Michael L. Freaney, Woochan Kim, Yeona Kang, Dardo Tomasi, Christopher Johnson, Chung-An Fang, Nora D. Volkow, Sung Won Kim

**Affiliations:** 1 Laboratory of Neuroimaging, National Institute On Alcohol Abuse and Alcoholism, National Institutes of Health, Bethesda, MD 20892 USA; 2 Department of Mathematics, Howard University, Washington, DC 20059 USA; 3 Molecular Imaging Branch, National Institute of Mental Health, National Institutes of Health, Bethesda, MD 20892 USA

**Keywords:** Neuroscience, Pharmacology

## Abstract

[^11^C]Carfentanil ([^11^C]CFN) is the only selective carbon-11 labeled radiotracer currently available for positron emission tomography (PET) imaging of mu opioid receptors (MORs). Though used extensively in clinical research, [^11^C]CFN has not been thoroughly characterized as a tool for preclinical PET imaging. As we were occasionally observing severe vital sign instability in rat [^11^C]CFN studies, we set out to investigate physiological effects of CFN mass and to explore its influence on MOR quantification. In anesthetized rats (n = 15), significant dose-dependent PCO_2_ increases and heart rate decreases were observed at a conventional tracer dose range (IV, > 100 ng/kg). Next, we conducted baseline and retest [^11^C]CFN PET scans over a wide range of molar activities. Baseline [^11^C]CFN PET studies (n = 27) found that nondisplaceable binding potential (BP_ND_) in the thalamus was positively correlated to CFN injected mass, demonstrating increase of MOR availability at higher injected CFN mass. Consistently, when CFN injected mass was constrained < 40 ng/kg (~ 10% MOR occupancy in rats), baseline MOR availability was significantly decreased. For test–retest variability (TRTV), better reproducibility was achieved by controlling CFN injected mass to limit the difference between scans. Taken together, we report significant cardiorespiratory depression and a paradoxical influence on baseline MOR availability at conventional tracer doses in rats. Our findings might reflect changes in cerebral blood flow, changes in receptor affinity, or receptor internalization, and merits further mechanistic investigation. In conclusion, rat [^11^C]CFN PET requires stringent quality assurance of radiotracer synthesis and mass injected to avoid pharmacological effects and limit potential influences on MOR quantification and reproducibility.

## Introduction

Mu opioid receptors (MORs) are widely expressed throughout the nervous system and play a central role in modulating pain and reward. Over the past decade, opioid research has become a national priority in the United States due to the surge in overdose deaths attributed to synthetic opioids, which activate MORs, particularly fentanyl and its analogs^1^. Among synthetic opioids, carfentanil (CFN) is one of the most dangerous; ironically, its isotopologue, radioactive [^11^C]CFN is the sole valuable clinical research tool for selectively measuring brain MORs with positron emission tomography (PET)^2^. [^11^C]CFN PET has provided insights into MOR-mediated mechanisms underlying reward^3^, craving and relapse^4,5^, pain^6^, behavioral activation^7^, obesity^8^, and the actions of MOR drugs through direct competition studies^9^.

Since CFN was first radiolabeled by Dannals et al. in 1985^10^, [^11^C]CFN has been well-characterized for clinical research PET^11^. However, even at microgram doses in humans (25–69 ng/kg), Minkowski et al. raised safety concerns, showing 60% of healthy subjects experienced at least one adverse effect, most commonly dizziness^12^. Due to its high potency (K_D_ = 0.08 nM^13^) and toxicity (LD_50_ = 3.13 mg/kg^14^), radiosynthesis of [^11^C]CFN requires extensive efforts to achieve high molar activity, ensuring sub-pharmacological doses in human PET studies (≤ 30 ng/kg)^15^. In turn, this strict constraint for [^11^C]CFN minimizes MOR occupancy by carbon-12 and undesirable physiological responses from its pharmacological effects.

Alongside clinical PET imaging, small animal PET provides greater experimental flexibility with higher throughput for translational studies. Despite these scientific advantages, however, only five rat [^11^C]CFN PET studies have been published compared to more than one hundred human [^11^C]CFN studies. Furthermore, to our knowledge, no studies to date have systematically characterized the reproducibility of [^11^C]CFN PET in rats related to molar activity. While conducting baseline MOR measurements in anesthetized rats, we observed significant cardiac and respiratory depression using conventional [^11^C]CFN injected doses. Herein, we report cardiorespiratory responses in rats after administering three conventional tracer level doses of CFN. Furthermore, we investigate the potential influence of CFN injected dose on MOR quantification along with reproducibility using a wide range of [^11^C]CFN molar activities.

## Results

### Respiratory and cardiac depression after intravenous carfentanil administration

**Figure 1:**
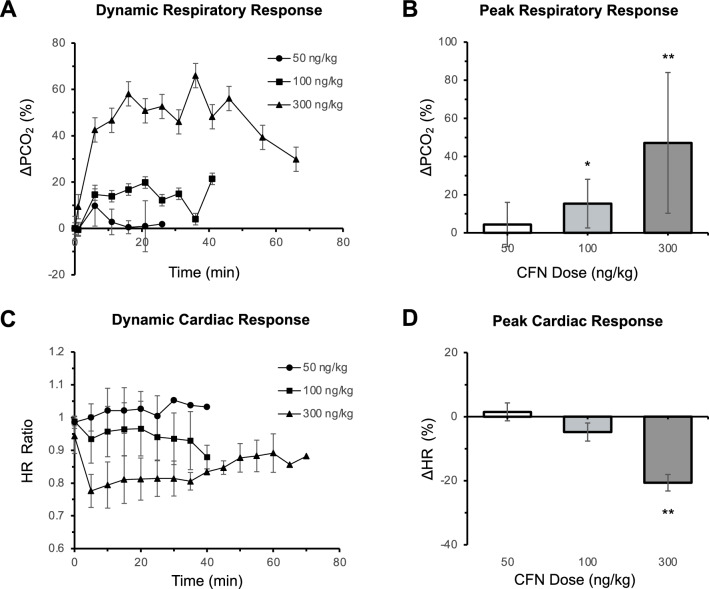
Physiological effect of CFN at 50, 100, and 300 ng/kg. (**A**) Dynamic blood PCO_2_ levels significantly increased at higher doses, indicating CFN induced respiratory depression. Arterial blood PCO_2_ defined as percent change from baseline (n = 3 at 50 ng/kg, n = 6 at 100 and 300 ng/kg). (**B**) Peak respiratory response defined as the average percent change from baseline between t = 5 to t = 15 min after CFN injection. (**C**) Dynamic HR change from baseline over time at 50 (n = 3), 100 (n = 6), and 300 ng/kg (n = 6). (**D**) Peak cardiac response defined as the average percent change from baseline between t = 5 to t = 15 min after CFN administration. All values reported as mean ± SEM. Significance reported using one-sample one-tailed Student’s *t*-test (*p < 0.05, **p < 0.01, ***p < 0.001).

For dynamic respiratory depression quantification, the partial pressure of carbon dioxide (PCO_2_) was measured in arterial blood. Baseline PCO_2_ measurements before intravenous (IV) CFN administration ranged from 66 to 72 mmHg (n = 15). CFN gradually increased PCO_2_, peaking at 6–21 min for 100 ng/kg, and 11–26 min at 300 ng/kg (Fig. 1A and Table S1). CFN induced respiratory depression was dose-dependent; in detail, PCO_2_ fluctuation from baseline was 4.4 ± 11.6% at 50 ng/kg (n = 3), and increased 15.3 ± 12.8% at 100 ng/kg (n = 6) and 47.1 ± 36.9% at 300 ng/kg (n = 6) (mean ± SEM, Fig. 1B). PCO_2_ increase from baseline was significantly different for 100 ng/kg (p = 0.02) and 300 ng/kg (p = 0.003), but not for 50 ng/kg (p = 0.13, one-sample t-test). Compared with vehicle group (n = 3), no significant PCO_2_ change was observed for 50 ng/kg (data not shown).

For cardiac depression, monitored heart rate fluctuated from baseline after 100 ng/kg (−4.82 ± 2.83%, n = 6) and 50 ng/kg (1.49 ± 2.79%, n = 3) CFN administration, although these changes were not statistically significant (Fig. 1C, Table S2). At 300 ng/kg, significant cardiac depression was observed (−20.6 ± 2.6%, n = 6; p = 0.004), as shown in Fig. 1D.

### Radiosynthesis of [^11^C]Carfentanil

The total synthesis time of [^11^C]carfentanil was 40 min. Decay-corrected radiochemical yield and purity was 40–72% and > 99%, respectively. Molar activity of synthesized [^11^C]CFN at the time of injection was 140.6–677.9 GBq/µmol.

### Baseline MOR availability measured by [^11^C]Carfentanil PET

**Figure 2:**
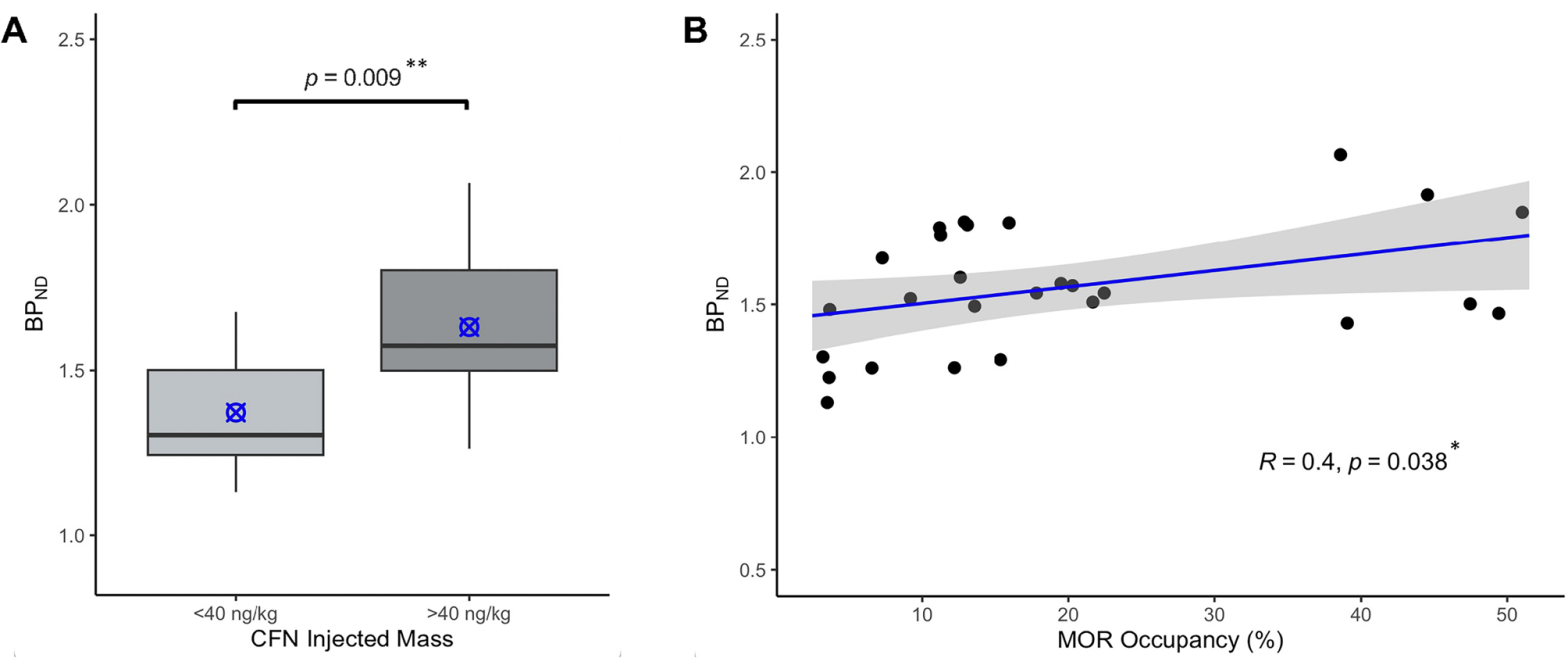
CFN injected mass influence on baseline (scan 1) [^11^C]CFN BP_ND_ estimation. (**A**) Mean BP_ND_ measured when CFN injected dose is constrained below 40 ng/kg (10% MOR occupancy) is significantly lower than BP_ND_ obtained at higher masses (p = 0.009, unpaired Student’s *t*-test). Box plots represent median, 25th, and 75th percentiles and blue markers represent mean values of each group (n = 7 vs 20). (**B**) MOR binding sites (%) occupied by CFN injected mass is significantly associated with baseline BP_ND_ (p = 0.038). Points represent individual subject data (n = 27) and light gray shaded area corresponds to the line best fit with a 95% confidence interval (R = 0.4, Pearson correlation). MOR occupancy was estimated using Eq. 1.

MOR availability was quantified at baseline (scan 1, n = 27) as nondisplaceable binding potential (BP_ND_) in the thalamus, a region of high MOR density, using the cerebellum as a reference region. [^11^C]CFN PET was performed over a wide range of molar activities by the addition of carbon-12 CFN prior to injection. The molar activity of formulated [^11^C]CFN varied from 17.3–678 GBq/µmol at time of injection, resulting in CFN injected mass from 13.1–411 ng/kg (Table S3, Scan 1). The average baseline BP_ND_ (scan 1) was 1.56 ± 0.23 (coefficient of variation [CV] = 15%) and varied from 1.13–2.07. Additionally, when the CFN injected mass was less than 40 ng/kg (10% MOR occupancy), the mean BP_ND_ was 1.37 ± 0.19 (n = 7, CV = 14%). At CFN injected doses greater than 40 ng/kg, BP_ND_ was 1.63 ± 0.21 (n = 20, CV = 13%) as shown in Fig. 2A. This pronounced increase in MOR availability was significant (p = 0.009, Student’s *t*-test).

Across all the subjects, baseline MOR availability was significantly associated with MOR occupancies of administered CFN (Fig. 2B). Specifically, MOR occupancy produced by CFN injected mass is positively correlated with measured BP_ND_ (R = 0.4, p = 0.038, Pearson correlation). BP_ND_ at high CFN injected doses (> 100 ng/kg) was 1.70 ± 0.27 (n = 6) and increased 24% from mean BP_ND_ estimated below 40 ng/kg injected CFN (10% MOR occupancy).

### Test–retest variability of [^11^C]Carfentanil PET quantification

**Figure 3:**
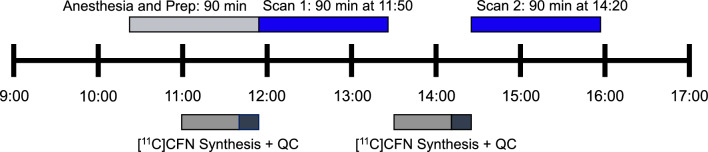
Schematic representations of [^11^C]CFN test–retest PET experiments. Middle timeline represents time of day for single PET experiment. Above the timeline represents time blocks for animal procedures and PET imaging. Below the timeline represents time blocks for [^11^C]CFN synthesis and quality control.

**Figure 4:**
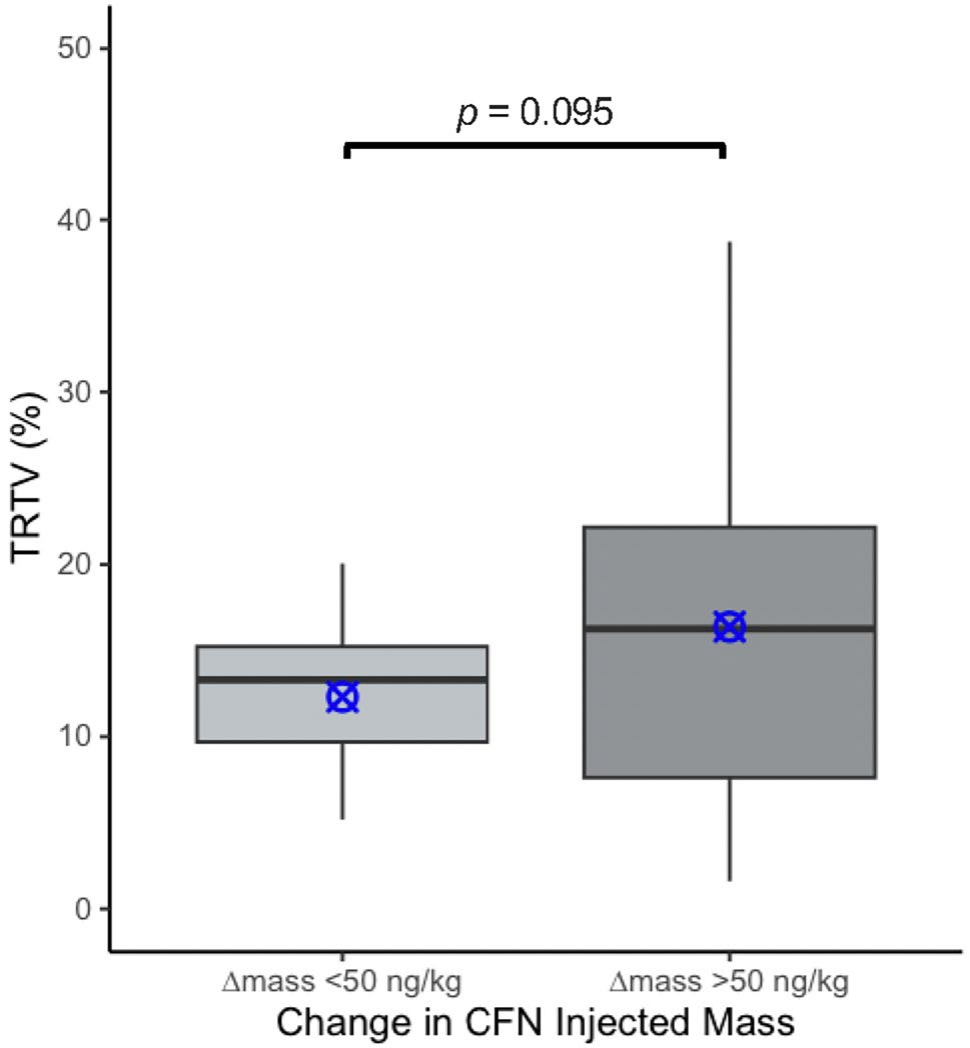
Test–Retest Variability of [^11^C]CFN BP_ND_. Mean TRTV decreased by 4.08% but was not considered significant (p = 0.095, one-tailed Student’s *t*-test). Box plots represent median, 25th, and 75th percentiles and blue markers represent mean values of each group (n = 12 vs 15).

All rats (n = 27) consecutively underwent a retest session (scan 2) at 2.5 h following scan 1 to provide the test–retest variability (TRTV) under anesthesia. A schematic representation of test–retest PET experiments is illustrated in Fig. 3. Average [^11^C]CFN BP_ND_ at retest (scan 2) was 1.52 ± 0.24 (CV = 16%), consistent with scan 1, and ranged from 1.03–2.03. The TRTV of [^11^C]CFN BP_ND_ was 14.58 ± 8.46% (CV = 58%) and the test–retest difference varied considerably from 1.6–39%. When both test and retest scans were conducted below 40 ng/kg CFN mass (10% MOR occupancy), TRTV was 15.47 ± 0.24% (n = 3). Apparently, comparable CFN mass difference between scans (Δmass < 50 ng/kg, n = 12) showed in an improved trend of reproducibility (averaged TRTV = 12.31 ± 4.68%) with low variation, while inconsistent doses (Δmass > 50 ng/kg, n = 15) increased averaged TRTV (16.39% ± 10.39%) with very high variation (p = 0.095, Student’s *t*-test) (Fig. 4).

## Discussion

This report documents that a conventional radiotracer dose of [^11^C]CFN induced unexpected physiological responses in anesthetized rats and altered MOR quantification in PET. To our knowledge, the injection limit of CFN mass in clinical PET studies has been set to 30–50 ng/kg^16^ from which an injection limit for rat studies corresponds to an estimated 192–310 ng/kg using the human equivalent dose (HED) guideline^17^. Previously, [^11^C]CFN PET studies have administered > 200 ng/kg CFN mass to rats^18^, while other studies did not report information regarding mass injected^19,20^. Recently, two additional rat [^11^C]CFN PET studies constrained the injected mass to below 100 ng/kg^21^ and 30 ng/kg^22^, respectively. In general, FDA mandates quality analysis prior to tracer administration in clinical PET studies despite the short half-life of C-11 (20.4 min), which allows one to tightly control the injection mass of [^11^C]CFN for human safety. However, it is not required in preclinical studies as molar activity decreases during radiochemical analysis. Based on our results, determination of CFN mass dose administered to rats would be desirable to increase the reliability and quality of the PET data. To achieve this, one practical option could be to approximately estimate the CFN mass during radiotracer purification.

Incidentally, we acquired [^11^C]CFN PET data over a wide molar activity range due to fluctuations of C-12 mass in our new radiochemistry laboratory and, in some instances, observed severe respiratory depression. Since such a response had not been reported, we conducted a series of controlled studies to investigate the physiological effects of IV CFN over an upper (300 ng/kg), middle (100 ng/kg), and lowest (50 ng/kg) range in anesthetized rats. Significant respiratory depression was observed at CFN doses > 100 ng/kg, while 50 mg/kg CFN did not significantly alter baseline PCO_2_ levels (n = 3) in anesthetized rats (Fig. 1). This is consistent with a fundamental premise of PET; that is, to administer radiotracers at sub-pharmacological doses that do not induce any pharmacological effect. Therefore, our findings suggest the need to optimize molar activity and apply stringent quality assurance when conducting [^11^C]CFN PET studies in rats.

In addition, we investigated baseline [^11^C]CFN BP_ND_ broadly over this injected mass range (13.1–411 ng/kg) and report a significant influence between CFN injected mass and in vivo MOR availability (Fig. 2). As molar activity decreases, the proportion of carbon-12 to carbon-11 radiotracer increases, thereby reducing the number of available receptors by self-blocking and typically leading to a decrease in BP_ND_^23^. Paradoxically, our results show the opposite effect, so that CFN doses > 100 ng/kg increased MOR availability 24% compared to negligible doses (< 40 ng/kg, < 10% MOR occupancy, which was estimated by Eq. 1). Furthermore, despite statistical insignificance, averaged TRTV was exacerbated with high variation between repeated measures when the injected dose of CFN was inconsistent (Δmass > 50 ng/kg) (Fig. 4), indicating the mass dependance of [^11^C]CFN quantification. This could potentially be important in the cases where molar activity is expected to fluctuate between [^11^C]CFN scans of MOR availability.

While it is outside of the scope of this report, high brain uptake of [^11^C]CFN at high mass doses may be due to an increase of cerebral blood flow as carbon dioxide, which accumulates with respiratory depression, is a potent vasodilator^24^ (Fig. S1). Since SRTM is not sensitive to global changes in cerebral blood flow^25^, another possible cause may be region-specific changes in cerebral blood flow induced by CFN itself. Though CFN has not been tested, Wagner et al. showed that the opioid agonist remifentanil induced heterogeneous changes in regional cerebral blood flow in the human brain^26^. Alternatively, increased brain uptake could reflect molecular level changes such as receptor desensitization that may alter the ligand binding mode^27,28^. Lastly, receptor internalization/trafficking along with radiotracer may prolong residence time inside the target tissue due to trapping inside cells.

Overall, regardless of the mechanisms underlying the increased MOR availability observed with larger CFN injected doses, we recommend that the mass delivered to rats should be restricted below 40 ng/kg to avoid physiological influence on PET MOR measurements. Recent advances in preclinical PET scanners have improved their sensitivity allowing for quantitative PET measurements to be obtained with injected activities as low as 5 MBq. Using this guideline, [^11^C]CFN molar activity > 180 GBq/μmol can achieve CFN masses < 40 ng/kg in rats and limit the potential confounding effects of CFN mass on MOR quantification. However, in cases where high [^11^C]CFN molar activity is not feasible or true baseline MOR measurements are not necessary, we recommend to carefully report CFN injected mass and administer consistent doses to improve variability.

### Limitations

In our [^11^C]CFN PET studies, it is not possible to determine whether measured changes in BP_ND_ are due to differences in MOR density, [^11^C]CFN binding affinity, or competition with endogenous opioid peptide tone. Furthermore, isoflurane anesthesia remains a confounding factor that may contribute significantly to inter-subject variability of physiological effects and PET MOR measurements. When comparing dose–response relationships between rodents and humans with [^11^C]CFN, it is important to note roles of anesthesia. This may shift the dose–response curve of CFN to cause greater physiological effects since isoflurane is known to cause cardiac and respiratory depression^29,30^. Finally, the MOR occupancy produced by CFN injected mass was calculated using the analgesic ED_50_ of IV CFN in rats, but the true receptor occupancy could differ.

## Conclusion

To the best of our knowledge, we report for the first time cardiac and respiratory depression following [^11^C]CFN injection at tracer level doses that influence MOR quantification. This may be particularly important for MOR occupancy studies of opioid agonist drugs, since there can be additive effects that result in cardiorespiratory depression^31^. Thus, a new selective MOR antagonist radiotracer needs to be developed, which would remove potential pharmacological effects regardless of injected mass. Furthermore, a fluctuation of injected mass would exacerbate reproducibility between scans within the same subject. Our results indicate that [^11^C]CFN PET in rodents requires scrupulous experimental design and validation.

## Materials and methods

### General

All animal handling and experiments were approved by the Animal Care & Use Committee (ACUC) at the National Institutes of Health (MIB-03, NIAAA 19-01) in compliance with guidelines of Animal Welfare Act Regulations and Public Health Service Policy on Humane Care and Use of Laboratory Animals of the USA. Male Long Evans rats were purchased from Charles River Laboratories (Frederick, MD) and were housed 2–3 per cage with wood chip bedding under a reverse 12 h/12 h light/dark cycle. Food and water were provided ad libitum. Isoflurane (Forane, 99.9%) in 100% O_2_ carrier gas was used as anesthetic during surgery, [^11^C]CFN PET scans, and respiratory depression experiments. Tubing for catheters (BTPE-10 for infusion, BTPU-27 for blood withdrawal) and other surgical materials were obtained from Instech Laboratories (Plymouth Meeting, PA). During PET imaging, heart rate (HR), respiratory rate (RR), and spO_2_ were monitored and recorded for at least one subject/scan with a MouseSTAT Pulse Oximeter or Heart Rate Module PhysioSuite (Kent Scientific, Litchfield, CT). Body temperature was maintained using a heat lamp as an external heat source (Brant Industries, Bronx, NY). Intravenous infusion of [^11^C]CFN or CFN was performed by a programmable dual syringe pump (Harvard Apparatus, Holliston, MA). Blood gases were analyzed using an OPTI CCA-TS2 Blood Gas and Electrolyte Analyzer with E-Glucose cassettes (OPTI Medical, Roswell, GA). PET data were analyzed using PMOD v3.807 (PMOD Technologies Ltd, Zurich, Switzerland). The study was carried out in compliance with the Animal Research: Reporting of In Vivo Experiments (ARRIVE) guidelines.

### Cardiac and respiratory depression experiments

Rats (n = 15, 321.4 ± 36.2 g) were anesthetized in a chamber filled with 5% isoflurane. Anesthesia was then maintained to effect using 1.5–2.5% isoflurane in a stream of oxygen gas (1.5 L/min), measuring HR throughout each study. A femoral vein catheter was placed for CFN injection, and a femoral arterial catheter was placed to collect samples for blood gas assessment. All the sample collection syringes were heparinized before use. Three baseline samples (200 µL) were collected 5 min apart to ensure steady state of baseline PCO_2_ level, after which time CFN (50, 100, or 300 ng/kg) was administered as a bolus (500 µL) over 1 min using the syringe pump. Blood samples (200 µL) were collected at 1 min post CFN injection and every 5 min thereafter for up to 70 min. Each blood sample was assessed immediately after collection.

Baseline HR was defined as the average value of the 5 min period prior to CFN injection. Raw data was smoothed using a moving average filter (window = 6 s) and resampled from 1 Hz to 1/30 Hz. For arterial blood gas analysis, three baseline measurements were averaged and used as baseline data. HR and blood data were normalized to baseline, and the dynamic and peak effect for PCO_2_ and HR was expressed as percent change from baseline or ratio to baseline, respectively.

### Radiosynthesis of [^11^C]Carfentanil

The starting material of [^11^C]CFN synthesis, 4-[(1-oxopropyl)phenylamino]1-(2-phenylethyl)-4-piperidinecarboxylic acid was purchased from American Biochemicals (College Station, TX). A solution of non-radioactive CFN was obtained from prior [^11^C]CFN production with poor molar activity after radiation decay. Its concentration was determined by analytical HPLC (µg/mL). All other chemicals including carfentanil reference (Cerilliant, Cat #: C-162) for quality control were purchased from Sigma-Aldrich (Darmstadt, Germany) and used without any further purification. Radioactive precursor, [^11^C]CH_3_I, was produced by TRACERlab FX-MeI (GE Healthcare Technologies, Aurora, OH) using [^11^C]CO_2_ generated by a cyclotron (GE PETtrace 800, GE Healthcare, Aurora, OH). [^11^C]CFN was synthesized using a TRACERlab FX-M (GE Healthcare Technologies, Aurora, OH, USA). Preparative HPLC was conducted using an isocratic solvent consisting of 48.5% ethanol in 0.01 M phosphate buffer (pH 7.2–7.4) using a semi-preparative HPLC column (Chromolith semiprep RP-18E, 10 × 100 mm; Sigma-Aldrich, Saint Louis, MO). Quality control was performed via analytical HPLC using a ZORBAX Eclipse XDB-C18 column (80°A, 4.6 × 150 mm, 5 µm; Agilent, Wilmington, DE). The analytical HPLC employed an isocratic solvent consisting of 40% acetonitrile : 60% aqueous 0.1% trifluoroacetic acid solution.

Synthesis of [^11^C]CFN and quality control were carried out using methods described previously with minor modifications to achieve higher molar activity^10,32^. Notably, a N_2_/O_2_ mixture was bombarded with a 50 µA proton beam for 50 min in a cyclotron to produce [^11^C]CO_2_, which was trapped in a stainless steel tubing cooled in liquid argon and released to [^11^C]CH_3_I production in the GE module^33^. Prior to the main production, an additional bombardment (5 µA, 5 min) was often performed to maximize molar activity.

### PET imaging experiments

Baseline PET scans for 27 rats (311 ± 36.2 g) were performed with [^11^C]CFN across two different PET imaging systems due to the replacement of a Siemens MicroPET Focus 220 (F220) system with new a Mediso Ltd. LFER 150 PET/CT system in the laboratory. In 19 rats, dynamic [^11^C]CFN PET scans were acquired on the former, while an additional 8 rats were scanned on the latter. For all subjects, a similar preparation protocol was followed. Briefly, anesthesia was induced (5% isoflurane for 5 min) and then maintained at 1–2% for the duration of scanning. An intravenous catheter was placed for radiotracer injection and [^11^C]CFN was administered as a 1 min bolus (9.85 ± 4.92 MBq) and immediately flushed with heparinized saline (250 µL). PET data was acquired in list-mode for 80 min and binned into 23 frames (6 × 20 s, 5 × 60 s, 4 × 120 s, 3 × 300 s, 3 × 600 s, and 1 × 1200 s). All subjects underwent two [^11^C]CFN PET scans 2.5 h apart (baseline (test) and retest) without moving in the scanner bore and the total time under anesthesia did not exceed 6 h.

Dynamic PET data acquired on the F220 system was reconstructed over a 350–650 keV energy window and 6 ns timing window. Prior to PET imaging, a 10 min transmission scan with a Co-57 point source was performed for attenuation correction of PET data. Images were reconstructed from 3D sinograms using 2D filtered-back projection. For PET data acquired on the LFER 150 PET/CT system, a computed tomography (CT) scan was first acquired for CT-based attenuation correction of PET data. Corrected PET data was then reconstructed over a 400–600 keV energy window and 5 ns timing window in 3D mode using a version of the ordered subset expectation maximization (OSEM) algorithm (Tera-Tomo 3D) provided with the scanner software (Nucline, Mediso ltd.) with 2 iterations of 9 subsets. There were no significant differences in [^11^C]CFN BP_ND_ measured between the two imaging systems (mean BP_ND_ = 1.57 vs 1.56, n = 19 vs 8, p = 0.931, Student’s t-test, SI Fig. 2).

### PET image analysis

A set of [^18^F]FDG and [^11^C]CFN PET scans of the same rat was obtained in sequence to build up an in-house template for F220 imaging, ensuring no movement between two scans. [^18^F]FDG images were automatically registered to the rat FDG template provided in PMOD^34^ and the resulting transformation matrix was applied to dynamic [^11^C]CFN PET images. For subjects imaged on the LFER 150 system, CT images acquired in the same FOV were used for automatic alignment of dynamic [^11^C]CFN PET images into the same rat atlas space. For all subjects, alignment was performed using a normalized mutual information dissimilarity function, 0.8 mm sampling rate, and Powell’s minimization method. The VOI atlas associated with the template image set (T2 weighted MRI, CT, [^18^F]FDG PET) was then applied to each subject to generate time-activity curves normalized to standard uptake value (SUV, g/mL). Two regions of interest (ROIs) were analyzed for [^11^C]CFN uptake: the thalamus due to its high concentration of MORs^35^ and the cerebellum as a reference region due to its minimal MOR expression^28^. Binding potential with reference to nondisplaceable binding (BP_ND_) was determined using the Simplified Reference Tissue Model (SRTM) as described previously^36^.

MOR occupancy produced by CFN injected mass was determined using a conventional equation (Eq. 1) first described by Hume et al. 1998^37^:

1 $${{Occ}} = \frac{Act}{Wt E{D}_{50} SpAct+Act} \times 100\%$$

where Act is the injected dose (MBq), SpAct is the molar activity at TOI (MBq/nmol), Wt is subject body weight (kg), and ED_50_ is the dose (nmol/kg) to reduce maximum radioligand binding by 50%. For CFN, only the analgesic ED_50_ has been reported for intravenous administration in rats. Therefore, this value (0.37–0.44 μg/kg)^14^ was used to estimate the CFN dose required to reduce maximal MOR binding by 50%.

Test–retest variability was determined as using the following equation (Eq. 2), where BP_ND,1_ is the test measurement and BP_ND, 2_ is the retest measurement:

2 $$TRTV= \frac{|{BP}_{ND, 1}-{BP}_{ND, 2}|}{\frac{1}{2}({BP}_{ND, 1}+{BP}_{ND, 2})} \times 100\%$$

### Statistics

Results are reported as mean ± standard deviation unless otherwise noted. Student’s *t*-tests were performed on group means and statistical significance was determined by p-values < 0.05. For cardiac and respiratory depression experiments, one-sample one-tailed t-tests were performed and the hypothetical mean was zero change from baseline values. For baseline [^11^C]CFN comparisons, two-sample two-tailed t-tests were performed. For comparison of [^11^C]CFN TRTV, two-sample one-tailed t-test was performed. The relationship between CFN injected mass and [^11^C]CFN BP_ND_ was determined using Pearson correlation in RStudio (v 1.1.383).

### Supplementary Information

Supplementary Information.

## Data Availability

The datasets analyzed during the current study are available in the supplementary information or from the corresponding authors upon reasonable request.
