# Supplementary material for: Investigation of [11C]carfentanil for mu opioid receptor quantification in the rat brain
Source: Sci Rep. 2024 Jul 15;14:16250. doi: 10.1038/s41598-024-66144-4 (PMC11250808; doi:10.1038/s41598-024-66144-4)
Supplement: Supplementary file 1 — Supplementary Information. [file 41598_2024_66144_MOESM1_ESM.doc]

**Supplement Table 1.**

| Time (min) | 50 ng/kg (n=3) | | 100 ng/kg (n=6) | | 300 ng/kg (n=6) | |
| --- | --- | --- | --- | --- | --- | --- |
|  | Mean | SD | Mean | SD | Mean | SD |
| 0 | 0 | 0 | 0 | 0 | 0 | 0 |
| 1 | -0.38 | 3.03 | -0.52 | 3.94 | 9.37 | 7.25 |
| 6 | 9.83 | 8.83 | 14.60 | 9.64 | 42.53 | 21.63 |
| 11 | 2.81 | 5.53 | 13.90 | 14.05 | 46.68 | 22.96 |
| 16 | 0.51 | 2.80 | 16.79 | 14.58 | 58.08 | 19.49 |
| 21 | 0.96 | 10.99 | 19.80 | 16.50 | 50.76 | 17.93 |
| 26 | 1.91 |  | 12.20 | 7.15 | 52.64 | 19.07 |
| 31 |  |  | 14.97 | 22.42 | 45.99 | 19.61 |
| 36 |  |  | 4.00 |  | 65.96 |  |
| 41 |  |  | 21.38 | 18.89 | 48.21 | 19.40 |
| 46 |  |  |  |  | 56.07 |  |
| 56 |  |  |  |  | 39.30 |  |
| 66 |  |  |  |  | 29.883 |  |

**Table S1:** The dynamic percent change in PCO_2_ measured in arterial blood after intravenous administration of 50 ng/kg (n=3), 100 ng/kg (n=6), and 300 ng/kg (n=6) CFN. Values reported as mean and standard deviation (SD) and expressed as a percent. Means reported without SD represent n=1 for that time point.

**Supplement Table 2.**

| Time (min) | 50 ng/kg (n=3) | | 100 ng/kg (n=6) | | 300 ng/kg (n=6) | |
| --- | --- | --- | --- | --- | --- | --- |
|  | Mean | SD | Mean | SD | Mean | SD |
| 0 | 0.98 | 0.02 | 0.98 | 0.01 | 0.94 | 0.05 |
| 5 | 1.00 | 0.04 | 0.93 | 0.07 | 0.77 | 0.05 |
| 10 | 1.02 | 0.06 | 0.95 | 0.07 | 0.79 | 0.07 |
| 15 | 1.02 | 0.06 | 0.96 | 0.08 | 0.81 | 0.07 |
| 20 | 1.03 | 0.05 | 0.96 | 0.08 | 0.81 | 0.06 |
| 25 | 1.04 | 0.06 | 0.94 | 0.07 | 0.81 | 0.05 |
| 30 | 1.05 |  | 0.93 | 0.07 | 0.81 | 0.05 |
| 35 | 1.04 |  | 0.92 | 0.09 | 0.80 | 0.02 |
| 40 | 1.03 |  | 0.87 | 0.04 | 0.83 | 0.01 |
| 45 |  |  |  |  | 0.84 | 0.02 |
| 50 |  |  |  |  | 0.87 | 0.04 |
| 55 |  |  |  |  | 0.88 | 0.05 |
| 60 |  |  |  |  | 0.89 | 0.06 |
| 65 |  |  |  |  | 0.85 |  |
| 70 |  |  |  |  | 0.88 |  |

**Table S2:** The dynamic change in heart rate after intravenous administration of 50 ng/kg (n=3), 100 ng/kg (n=6), and 300 ng/kg (n=6) CFN. Values reported as mean and SD, expressed as a ratio to baseline HR. Means reported without SD represent n=1.

**Supplement Table 3.**

| **Subject** | **Body Weight (g)** | **Inj. Radioactivity (MBq)** | | **Molar Activity (GBq/μmol)** | | **Mass Dose (ng/kg)** | | **BP_ND_** | |
| --- | --- | --- | --- | --- | --- | --- | --- | --- | --- |
|  |  | **Scan 1** | **Scan 2** | **Scan 1** | **Scan 2** | **Scan 1** | **Scan 2** | **Scan 1** | **Scan 2** |
| 1 | 312.6 | 13.7 | 11.1 | 347.8 | 236.8 | 49.6 | 58.9 | 1.79 | 1.46 |
| 2 | 356.2 | 13.9 | 11.7 | 140.6 | 185.0 | 109.1 | 69.9 | 1.51 | 1.36 |
| 3 | 372.5 | 16.7 | 16.4 | 185.0 | 296.0 | 95.5 | 58.6 | 1.58 | 1.36 |
| 4 | 327.0 | 17.5 | 16.2 | 185.0 | 296.0 | 114.2 | 66.0 | 1.54 | 1.46 |
| 5 | 375.8 | 15.1 | 16.5 | 185.0 | 296.0 | 85.6 | 58.4 | 1.54 | 1.37 |
| 6 | 272.8 | 11.3 | 9.6 | 410.7 | 651.2 | 39.9 | 21.2 | 1.52 | 1.31 |
| 7 | 253.8 | 13.2 | 13.1 | 410.7 | 651.2 | 50.0 | 31.2 | 1.76 | 1.57 |
| 8 | 359.1 | 10.2 | 5.8 | 192.1 | 402.0 | 58.2 | 15.7 | 1.81 | 1.56 |
| 9 | 354.8 | 9.8 | 7.0 | 192.1 | 402.0 | 56.8 | 19.3 | 1.60 | 1.69 |
| 10 | 299.5 | 18.7 | 5.6 | 398.5 | 397.4 | 61.9 | 18.5 | 1.49 | 1.60 |
| 11 | 289.4 | 20.9 | 6.1 | 398.5 | 397.4 | 71.6 | 21.0 | 1.29 | 1.07 |
| 12 | 317.8 | 7.9 | 4.3 | 677.9 | 349.3 | 14.4 | 15.4 | 1.13 | 1.31 |
| 13 | 332.6 | 7.5 | 4.4 | 677.9 | 349.3 | 13.1 | 14.8 | 1.30 | 1.54 |
| 14 | 300.0 | 15.7 | 9.9 | 347.8 | 29.6 | 59.3 | 393.5 | 1.80 | 1.53 |
| 15 | 328.2 | 15.9 | 9.7 | 347.8 | 29.6 | 54.8 | 377.3 | 1.26 | 1.49 |
| 16 | 282.8 | 11.9 | 10.0 | 222.0 | 18.5 | 74.9 | 754.5 | 1.81 | 1.84 |
| 17 | 355.4 | 14.8 | 14.9 | 51.8 | 321.9 | 317.1 | 51.4 | 1.92 | 1.51 |
| 18 | 316.6 | 14.8 | 12.8 | 51.8 | 25.9 | 356.5 | 613.8 | 1.50 | 1.41 |
| 19 | 352.2 | 12.6 | 12.6 | 140.6 | 7.4 | 100.4 | 1907.2 | 1.57 | 2.03 |
| 20 | 285.0 | 4.1 | 3.9 | 381.6 | 18.2 | 14.9 | 297.7 | 1.23 | 1.51 |
| 21 | 272.1 | 3.8 | 4.1 | 21.7 | 259.5 | 252.8 | 23.1 | 1.43 | 1.90 |
| 22 | 270.6 | 4.0 | 5.6 | 381.6 | 18.2 | 15.1 | 446.9 | 1.48 | 1.67 |
| 23 | 262.3 | 3.6 | 4.3 | 21.7 | 259.5 | 247.9 | 24.9 | 2.07 | 1.94 |
| 24 | 281.1 | 5.8 | 4.0 | 262.8 | 14.0 | 30.9 | 405.7 | 1.68 | 1.83 |
| 25 | 301.5 | 5.1 | 4.8 | 17.3 | 309.2 | 385.5 | 20.2 | 1.47 | 1.50 |
| 26 | 285.4 | 5.3 | 4.4 | 262.8 | 14.0 | 27.7 | 437.4 | 1.26 | 1.03 |
| 27 | 284.7 | 5.1 | 4.6 | 17.3 | 309.2 | 411.0 | 20.4 | 1.85 | 1.25 |

**Table S3:** Results of [^11^C]CFN PET experiments.Values rounded to tenth decimal. BP_ND_ calculated for thalamus using SRTM.

**Supplement Figure 1.**


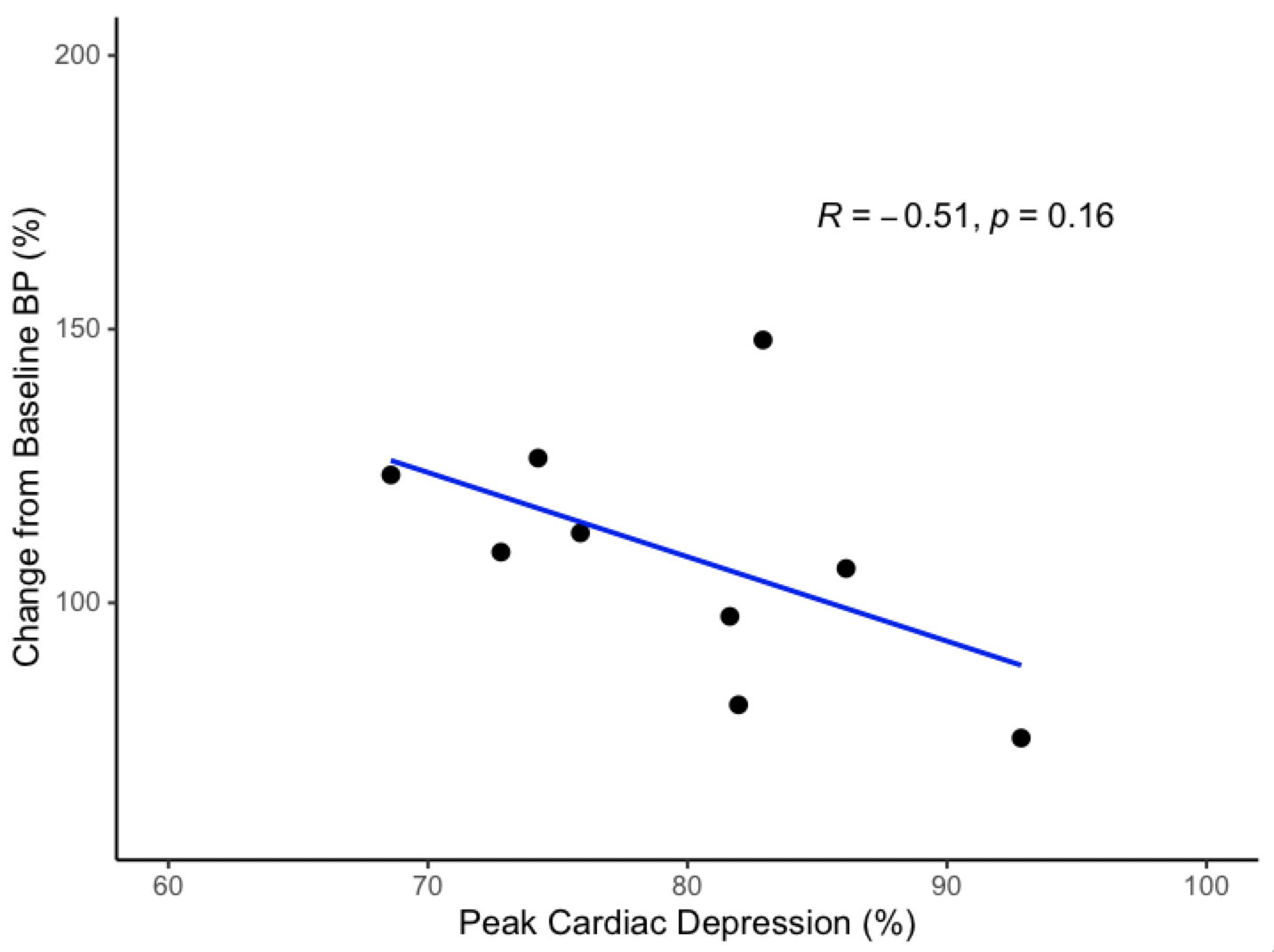


**Figure S1:** Increased [^11^C]CFN BP_ND_ relative to baseline is associated with stronger physiological change induced by CFN (p=0.16). Peak cadiac depression defined as the ratio of HR decrease to baseline beats/min before CFN administration. Points represent individual subject data (n=9) and blue line corresponds to the line best fit (R=-0.51, Pearson correlation).

**Supplement Figure 2.**


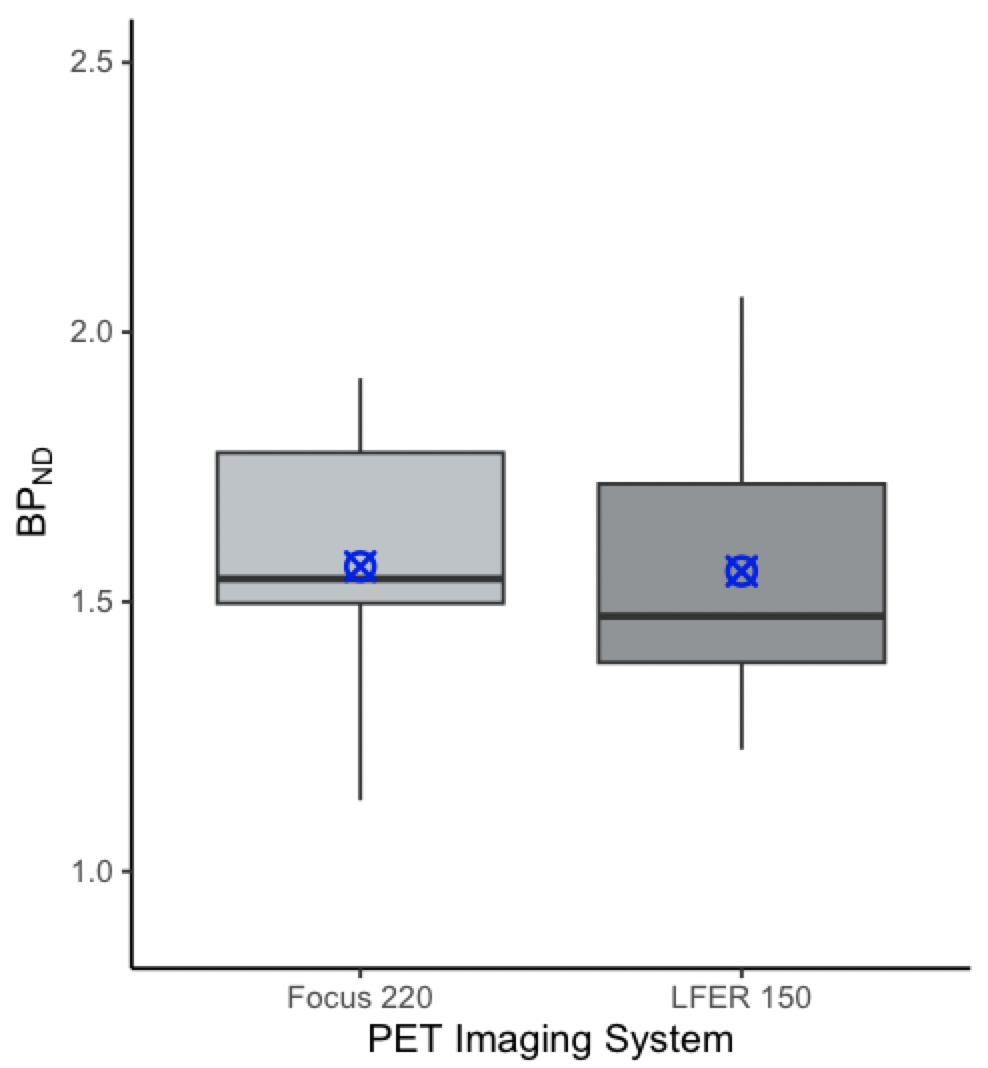


**Figure S2:** [^11^C]CFN BP_ND_ obtained using two different PET imaging systems, a Siemens Focus 220 or Mediso MultiScan™ LFER 150, respectively. Box plots represent median, 25^th^, and 75^th^ percentiles and blue markers represent mean values of each group (n=21 vs 8). There was no significant difference in baseline BP_ND_ quantification (p=0.931).
